# A decomposition of Fisher’s information to inform sample size for developing or updating fair and precise clinical prediction models for individual risk—part 1: binary outcomes

**DOI:** 10.1186/s41512-025-00193-9

**Published:** 2025-07-08

**Authors:** Richard D. Riley, Gary S. Collins, Rebecca Whittle, Lucinda Archer, Kym I. E. Snell, Paula Dhiman, Laura Kirton, Amardeep Legha, Xiaoxuan Liu, Alastair K. Denniston, Frank E. Harrell, Laure Wynants, Glen P. Martin, Joie Ensor

**Affiliations:** 1 Department of Applied Health Sciences, School of Health Sciences, College of Medicine and Health, University of Birmingham, Birmingham, UK; 2 National Institute for Health and Care Research (NIHR) Birmingham Biomedical Research Centre, Birmingham, UK; 3 Centre for Statistics in Medicine, Nuffield Department of Orthopaedics, Rheumatology and Musculoskeletal Sciences, University of Oxford, Oxford, OX3 7LD UK; 4 Cancer Research UK Clinical Trials Unit, Institute of Cancer and Genomic Sciences, College of Medical and Dental Sciences, University of Birmingham, Birmingham, UK; 5 Department of Biostatistics, Vanderbilt University School of Medicine, Nashville, TN USA; 6 Department of Epidemiology, Care and Public Health Research Institute (CAPHRI), Maastricht University, Maastricht, The Netherlands; 7 Department of Development and Regeneration, KU Leuven, Leuven, Belgium; 8 Division of Informatics, Imaging and Data Science, Faculty of Biology, Medicine and Health, University of Manchester, Manchester Academic Health Science Centre, Manchester, UK

**Keywords:** Clinical prediction models, Sample size, Uncertainty intervals, Instability, Classification, Fisher’s information matrix, Fairness

## Abstract

**Background:**

When using a dataset to develop or update a clinical prediction model, small sample sizes increase concerns of overfitting, instability, poor predictive performance and a lack of fairness. For models estimating the risk of a binary outcome, previous research has outlined sample size calculations that target low overfitting and a precise overall risk estimate. However, more guidance is needed for targeting precise and fair individual-level risk estimates.

**Methods:**

We propose a decomposition of Fisher’s information matrix to help examine sample sizes required for developing or updating a model, aiming for precise and fair individual-level risk estimates. We outline a five-step process for use before data collection or when an existing dataset or pilot study is available. It requires researchers to specify the overall risk in the target population, the (anticipated) distribution of key predictors in the model and an assumed ‘core model’ either specified directly (i.e. a logistic regression equation is provided) or based on a specified *C*-statistic and relative effects of (standardised) predictors.

**Results:**

We produce closed-form solutions that decompose the variance of an individual’s risk estimate into the Fisher’s *unit* information matrix, predictor values and the total sample size. This allows researchers to quickly calculate and examine the anticipated precision of individual-level predictions and classifications for specified sample sizes. The information can be presented to key stakeholders (e.g. health professionals, patients, grant funders) to inform target sample sizes for prospective data collection or whether an existing dataset is sufficient. Our proposal is implemented in our new software module *pmstabilityss*. We provide two real examples and emphasise the importance of clinical context, including any risk thresholds for decision making and fairness checks.

**Conclusions:**

Our approach helps researchers examine potential sample sizes required to target precise and fair individual-level predictions when developing or updating prediction models for binary outcomes.

**Supplementary Information:**

The online version contains supplementary material available at 10.1186/s41512-025-00193-9.

## Background

Studies developing or updating a clinical prediction model use a sample of data from a chosen target population (e.g. pregnant women; men diagnosed with prostate cancer) to produce a model for predicting an outcome value (e.g. birth weight) or estimating an outcome risk (e.g. 30-day mortality risk) in any individual from that target population. Models are created using approaches (e.g. regression) which map predictor information to outcomes at the individual level. An example is the ISARIC model [1], for use in hospitalised adults with suspected or confirmed COVID- 19 to estimate their risk of in-hospital clinical deterioration based on 11 predictors measured at hospital admission.

When developing or updating a model there is a responsibility to implement rigorous standards in study design and analysis [2–4], including study sample size. Many prediction model studies fail to include a justification of their sample size [5–12], despite being a recommendation in the TRIPOD statement [13], and in the recently updated TRIPOD + AI guidance [14]. However, sample size criteria have been proposed in recent years for model development [15–19]. In previous work for binary outcomes [16, 17], we outlined how to calculate the minimum sample size needed for model development based on (i) estimating the overall event risk precisely and (ii) minimising model overfitting for a regression-based prediction model in terms of overall fit and population-level calibration slope. This criteria aims to target a more reliable prediction model at least at the *population level*, corresponding to the first two model stability levels defined by Riley and Collins [20].

However, even when meeting this minimum sample size, the uncertainty in a model’s *individual-level *predictions can still be large. Individual-level predictions are a function of all the parameters (e.g. intercept, predictor effects) in the developed model, and large uncertainty of the parameter estimates leads to concerns of model instability [20–22], with subsequently wide uncertainty intervals (e.g. 95% confidence or credible intervals) around individual estimated risks. Models that exhibit unacceptably high levels of uncertainty in their estimated risks should not be recommended for use in individuals, as point estimates of risk might misinform treatment and other healthcare decisions. Thus, further sample size calculations would be helpful to address precision of risk estimates from prediction models at the individual level, not just population level. Such calculations would also help improve *fairness *of prediction models [23], such that the reliability (accuracy) of predictions is expected to be acceptable for all patient groups, including minoritised and underserved groups, not just in the population as a whole [24].

In this article, focusing on regression for binary outcomes, we propose a decomposition of Fisher’s information matrix to help researchers examine sample sizes required to obtain sufficiently precise individual-level predictions and classifications, corresponding to the third and fourth stability levels of Riley and Collins [20]. We begin by summarising our existing sample size approach and the software module *pmsampsize* that implements it. Then, we outline our new proposal to examine sample size requirements to target precise and fair individual-level predictions. We apply our proposal to two real examples, one before data collection and one when an existing dataset is available. Finally, we conclude with discussion.

## Existing sample size approach to precisely estimate overall risk and minimise overfitting for a binary outcome

Our current approach calculates the minimum required sample size for prediction model development using regression [15–17], to meet the following criteria:

- Criterion (i): a precise estimate of the overall outcome risk
- Criterion (ii): small overfitting of predictor effects
- Criterion (iii): small optimism in apparent model fit

For brevity, details of the calculations are provided in Supplementary material S1 and our previous papers [15–17, 25]. The approach is implemented in the Stata or R module *pmsampsize* [26, 27], with the user needing to specify the overall outcome risk (prevalence) and the anticipated model performance (quantified by Cox-Snell *R-*squared (${R}_{\text{CS}}^{2}$), Nagelkerke *R*-squared (${R}_{\text{Nagelkerke}}^{2})$ or the *C*-statistic) in the target population and the number of candidate predictor parameters for the model. For example, later in the article we consider the development of a model to estimate the risk of foot ulcer based on three predictor parameters. Assuming a *C*-statistic of 0.77 and an overall outcome risk of 0.059 (based on previous studies [28]), then *pmsampsize* (available in Stata or R) calculates at least 453 participants (27 events) are needed to meet criteria (i) to (iii).

Also, later we consider the development of a model to estimate the risk of acute kidney injury based on nine predictor parameters. Assuming an overall risk of 0.174 and a *C*-statistic of 0.78 based on a previous study [29], the *pmsampsize* calculates that at least 511 participants (89 events) are required.

## Methods: a new approach using a decomposition of Fisher’s information matrix

Criteria (i) to (iii) of our existing sample size calculation aim for stable (and well-calibrated) model predictions at the population level [20], such that the overall risk is precisely estimated and overfitting is low. As these are population-level criteria, they provide a *minimum* sample size calculation for model development to estimate individual-level risks. However, to be further reassured when planning prospective data collection or assessing the suitability of an existing dataset, researchers may want further guidance about the potentially (much) larger sample size needed to target individual-level stability and fairness.

To address this, below, we now propose a five-step process which utilises simulated (synthetic) or existing data and, based on logistic regression, a decomposition of the variance of individual-level predictions into Fisher’s unit information matrix and total sample size. It can be implemented using our new *pmstabilityss* module in Stata (type: net from https://joieensor.github.io/pm-suite/ or see https://github.com/JoieEnsor). An *R* version is available at https://github.com/JoieEnsor/pmstabilityss. The approach is most applicable when extending or updating a previous model and when existing or pilot study data are available, as it requires the user to input information about anticipated case-mix distributions and predictor weights. It provides an initial target sample size to inform, for example, grant applications. We emphasise that once data are (being) collected and analyses underway, internal validation is still important to check model stability and performance, for example via learning curves and stability plots [20, 30].

### Five-step approach to calculating and examining individual-level prediction and classification uncertainty for specified sample sizes

#### Step (1): identify a core set of predictors

These core predictors are variables well-known (in the clinical setting of interest) to contribute important predictive information, for instance as identified from previously published models, systematic reviews of prognostic factors [31], or conversations with clinical experts. Though additional predictors might be considered in the actual model development, the set of core predictors (and their combinations of values therein) represent the user’s minimum for examining sample size and uncertainty of individual-level risks. Examples of core predictors include (i) age and stage of disease for cancer outcome prediction; (ii) age, cholesterol and SBP for cardiovascular disease prediction; and (iii) insensitivity to monofilament, foot pulse absence and history of previous ulcer or amputation for diabetic foot ulcer prediction [28]. When updating or extending an existing model, the core predictors would be at least those already in the existing model.

In addition, the core set may also include variables linked to fairness checks. For example, it may be important to ensure a developed model has sufficiently precise predictions for different demographic subgroups, such as age, sex and ethnicity or other protected characteristics. Ensuring these variables are included in the core predictor set can help to examine this, even if they are deemed to not be important predictors themselves.

#### Step (2): specify the joint distribution of the core predictors

The joint distribution of predictors that are included in the final prediction model impacts the standard errors of logistic regression parameter estimates and thus influences the width of uncertainty intervals around individual-level risk estimates. Hence, step (2) requires the user to specify the joint distribution of core predictors selected in step (1). How exactly the user can specify this will depend on what information is available, as follows:

- *Existing dataset (e.g. from e-health database, previous study, or pilot study) is already available for model developers*: here, the joint distributions is observed directly and so the user does not need to do anything in this step; further, the existing dataset can be used directly in subsequent steps where needed (e.g. in step (4) to derive the unit information matrix). See kidney injury example later.
- *Synthetic dataset available*, *such as when an existing dataset exists but is not yet available for model developers (e.g. access is conditional on funding success).* Then, the data holders could be contacted and asked to provide a synthetic dataset that mimics the joint predictor distributions, for instance as obtained by a simulation-based approach, using packages such as *synthpop* in *R* [32]. A notable example is The Clinical Practice Research Datalink (CPRD), who have generated synthetic datasets to aid researchers improve workflows (https://www.cprd.com/synthetic-data).
- *Summary information available:* Sometimes, existing data holders could provide summary details of the joint distributions (e.g. cross-tabulations of categorical variables; variance–covariance matrix of continuous variables), to allow the user themselves to simulate a large synthetic dataset containing predictor values for, say, 10,000 individuals. See foot ulcer example later. Previous studies that already use the dataset of interest may summarise baseline variables (e.g. means, SDs, proportions) in their articles, for example within their baseline characteristics table. A challenge is that often, only the marginal distribution for each predictor will be summarised (from a published ‘table’ of baseline characteristics), and the correlation or conditional relationship amongst predictors will be unknown. In this situation, the impact of a range of different assumptions (e.g. from independence to strong correlations) could be examined (see kidney injury example later).

**Table 1:** Summary statistics for the expected precision and probability of misclassification for the AKI model when developed using 795 participants

**Ethnicity**	**95% uncertainty interval width:** *mean (min, max)*	**Probability of misclassification:** *mean (min, max)*
Asian	0.35 (0.03, 0.66)	0.19 (0, 0.50)
Black	0.24 (0.01, 0.67)	0.13 (0, 0.50)
Hispanic	0.29 (0.02, 0.76)	0.16 (0, 0.50)
Other	0.32 (0.02, 0.72)	0.19 (0, 0.50)
Unknown	0.21 (0.02, 0.63)	0.12 (0, 0.50)
White	0.13 (0.002, 0.71)	0.10 (0, 0,50)
*Overall*	0.17 (0.002, 0.76)	0.11 (0, 0.50)

#### Step (3): specify a ‘core model’ for how individual risks depend on core predictor values

Alongside an individual’s predictor values, the uncertainty around an individual’s risk estimate also depends on their risk estimate itself. Therefore, step (3) requires the user to specify a model that expresses how an individual’s risk depends on the values of core predictors from step (2). We refer to this as the ‘core model’. For example, one could specify a logistic regression model, such that an individual’s logit-risk is a function defined by an intercept and beta terms chosen to reflect the overall population risk and core predictor effects, respectively. This could be based on a previous model in the same field, and our applied examples illustrate this later. Also, the size and direction of beta coefficients could be based on previous studies or reviews of the predictive (prognostic) value of each factor, biological rationale or common sense. For example, for most situations, one could assume that age has a positive beta coefficient, as older ages increase risk; similarly, stage of disease or family history would have an assumed positive beta value.

The more accurate the ‘core model’, the more accurate the sample size calculation will be. In particular, it is most reliable in situations where an existing regression-based model is to be updated or extended, as then the existing model’s intercept and (relative) predictor weights can form the ‘core model’. In other situations, the ‘core model’ may be harder to specify; for example, predictor effects may not be readily available if previous models were unreported or ‘black box’. To address this, approximations are necessary based on other information:

- *Approach (a): specify the overall risk, C-statistic and relative weights of core predictors* *.* With this information, the approach based on Austin [33] can be used to identify a logistic regression equation that forms a ‘core model’ that adheres to the specified overall risk and *C*-statistic (equivalent to the area under the receiver operating characteristic curve, AUROC), whilst retaining the user’s chosen *relative* weight of core predictors. In brief, the approach simulates predictor values for a large number of participants (based on the distributions specified in Step (2)) and then an iterative process (based on Austin [33]) is used to identify values of the intercept ${(\alpha)}$ and a multiplicative factor ${(\delta)}$ of the following model:

1 $$\begin{aligned} & {y}_{i} \sim \text{ Bernoulli}\left({p}_{i}\right)\\ & \text{ln}\left(\frac{{p}_{i}}{1-{p}_{i}}\right)=\alpha +\delta \left({\beta }_{1}{x}_{1i}+{\beta }_{2}{x}_{2i}+\dots +{\beta }_{\text{P}}{x}_{\text{P}i}\right) \end{aligned}$$

where the beta coefficients are the relative weights specified by the user. Convergence is achieved when the model has reached the specified *C*-statistic and overall risk within a small margin of error.

- *Approach (b): specify the overall risk and C-statistic, whilst assuming the same weight of predictors after standardising continuous predictors.* This approach is akin to approach (a) but simplifies the process by assuming the ‘core model’ of Eq. (1) has equal weight of all predictors (i.e. all betas are set to 1) after standardising continuous predictors using their marginal mean and standard deviation from the joint distribution specified in Step (2) (e.g. uses $\left({x}_{1i}-{\bar{x}}_{1i}\right)/{\mathrm{SD}}_{x_{1i}}$ when ${x}_{1i}$ is a continuous variable, etc.). As in approach (a), the Austin method is then used to identify $\alpha$ and $\delta$ that ensures the ‘core model’ has a particular *C*-statistic and overall risk. Our kidney injury example illustrates this later.
  As mentioned, approaches (a) and (b) are pragmatic approaches to specify the ‘core model’ to enable researchers to examine potential sample sizes required for individual-level stability. Further examination of these approaches is needed; indeed, it is sensible to examine the impact of changing the assumptions (e.g. examine required sample size across a range of plausible *C*-statistic values) and does not negate the need for learning curves and stability plots once model development is underway [20, 30].

#### Step (4): derive Fisher’s unit information after decomposing Fisher’s information matrix

Steps (4) and (5) involve approximating (based on the information from steps (1) to (3)) the anticipated variance–covariance matrix ($$\text{var}(\widehat{\varvec{\upbeta}}))$$ of model parameter estimates ($$\widehat{{\varvec{\upbeta}}}={(\widehat{\alpha },\widehat{\beta }}_{1},{\widehat{\beta }}_{2},\dots ,{\widehat{\beta }}_{\text{P}}){\prime})$$ if we were to fit the assumed ‘core model’ for a specified sample size. This is needed, as the variance–covariance matrix subsequently dictates the variance of individual-level predictions. Step (4) begins by decomposing $$\text{var}(\widehat{\varvec{\upbeta}})$$ (i.e. the inverse of Fisher’s information matrix) into the total sample size ($$n)$$ and Fisher’s *unit* information matrix ($$\mathbf{I}$$):

2 $$\text{var}(\widehat{\varvec{\upbeta}})= {n}^{-1}{\mathbf{I}}^{-1}$$

where.

3 $$\mathbf{I}=E\left(\frac{\text{exp}\left({\mathbf{X}}{\prime}{\varvec{\upbeta}}\right)}{{\left(1+\text{exp}\left({\mathbf{X}}{\prime}{\varvec{\upbeta}}\right)\right)}^{2}}{\mathbf{X}}^{\prime}\mathbf{X}\right)=E\left(\mathbf{A}\right)$$

and $\mathbf{X}$ is the design matrix for the assumed ‘core model’, with each individual’s data corresponding to ${{\varvec{x}}}_{{\varvec{i}}}=(1,{x}_{1\text{i}},{x}_{2\text{i}},\dots ,{x}_{\text{Pi}})$.

The expected value ($E\left(\mathbf{A}\right))$ depends on the joint distribution of the predictors and the parameter values of the ‘core model’. A simple way to derive $E\left(.\right)$ is to calculate each of the components of $\mathbf{A}$ for each participant in the (existing or simulated) dataset from Step (2), using each participant’s predictor values combined with the logistic regression parameters from Step (3); then, the means (across all participants) of each component provides their expected values and forms $\mathbf{I}$.

For example, assuming a model with three core predictors, we can write:

4 $$\begin{aligned} \mathbf{I} &= E\left(\frac{\text{exp}\left(\alpha +\delta \left({\beta }_{1}{x}_{1i}+{\beta }_{2}{x}_{2i}+{\beta }_{3}{x}_{3i}\right)\right)}{{\left(1+\text{exp}(\alpha +\delta \left({\beta }_{1}{x}_{1i}+{\beta }_{2}{x}_{2i}+{\beta }_{3}{x}_{3i}\right)\right)}^{2}}{\mathbf{X}}^{\prime}\mathbf{X}\right)\\ & =E\left(\frac{\text{exp}\left(\alpha +\delta \left({\beta }_{1}{x}_{1i}+{\beta }_{2}{x}_{2i}+{\beta }_{3}{x}_{3i}\right)\right)}{{\left(1+\text{exp}(\alpha +\delta \left({\beta }_{1}{x}_{1i}+{\beta }_{2}{x}_{2i}+{\beta }_{3}{x}_{3i}\right)\right)}^{2}}\left[\begin{array}{cccc}1& {x}_{1i}& {x}_{2i}& {x}_{3i}\\ {x}_{1i}& {x}_{1i}^{2}& {x}_{1i}{x}_{2i}& {x}_{1i}{x}_{3i}\\ {x}_{2i}& {x}_{1i}{x}_{2i}& {x}_{2i}^{2}& {x}_{2i}{x}_{3i}\\ {x}_{3i}& {x}_{1i}{x}_{3i}& {x}_{2i}{x}_{3i}& {x}_{3i}^{2}\end{array}\right]\right)\\ &= E\left(\mathbf{A}\right)\end{aligned}$$

where $\mathbf{A}$ is a 4 by 4 matrix. Crucially, the parameters (i.e. $\alpha$, $\delta$ and all $\beta$ s) are replaced with their true values specified in Step (3) and the $x$ values are the (standardised or unstandardised) values of core predictors from Step (2). Then, to derive $E\left(\mathbf{A}\right)$, our software package calculates each of the 16 components of $\mathbf{A}$ for each participant in the existing or synthetic dataset, and the mean of each component (over all participants) provides their expected values and thus forms $\mathbf{I}$.

#### Step (5): examine the impact of sample size on the precision of individual risk estimates

The final step is to examine how sample size impacts the level of precision (uncertainty interval widths) around individual risk estimates. This is relevant when the user has an existing dataset (e.g. to ascertain if it is large enough) or when designing a new study with prospective data collection (to a priori identify the required sample size). These situations are now outlined as options A and B, below.

- Option A: calculate expected uncertainty of predictions for a given sample size (existing dataset)

Following the maximum likelihood theory of a logistic regression model, the variance of a predicted logit risk for a new individual is:

5 $${\text{var}}\left(\text{logit}({\widehat{p}}_{new})\right)={\text{var}}\left({{\varvec{x}}}_{new}\widehat{{\varvec{\upbeta}}}\right)={{\varvec{x}}}_{{{new}}} \, \text{var}(\widehat{\varvec{\upbeta}}){{\varvec{x}}}_{new}^{\prime}$$

where ${{\varvec{x}}}_{{{new}}}=(1,{x}_{1new},{x}_{2new},\dots ,{x}_{\text{P}new})$ are the predictor values for the new individual. Substituting in Eq. (2), this can be rewritten as:

6 $${\text{var}}\left(\text{logit}({\widehat{p}}_{new})\right)={n}^{-1}{{\varvec{x}}}_{{{new}}} {\mathbf{I}}^{-1}{{\varvec{x}}}_{new}^{\prime}$$

Subsequently, a 95% uncertainty interval around an individual’s estimated risk is,

7 $$\text{invlogit}\left[\text{logit}({\widehat{p}}_{new})\pm \left(1.96\times \sqrt{\text{var}\left(\text{logit}({\widehat{p}}_{new})\right)}\right)\right]$$

and substituting in Eq. (6) gives,

8 $$\text{invlogit}\left[\text{logit}({\widehat{p}}_{new})\pm \left(1.96\times \sqrt{{(n}^{-1}{{\varvec{x}}}_{new } {\mathbf{I}}^{-1}{{\varvec{x}}}_{new}^{\prime})}\right)\right]$$

where ‘invlogit’ is the inverse of the logit function (i.e. exp(${\widehat{p}}_{new}$)/(1 + exp(${\widehat{p}}_{new}$))).

Option A requires the user to apply Eq. (8) to participants from the target population, to derive uncertainty intervals conditional on a particular model development sample size ($n$) of interest. We already have the unit information matrix ($\mathbf{I}$) from Step (4), and the new participants can just be those from the existing or simulated dataset from Step (2) which already contains predictor values (${{\varvec{x}}}_{new}$). Each individual’s ${\widehat{p}}_{new}$ can be set to their ‘true’ risk defined by the ‘core model’ in Step (3). Thus, to apply Eq. (8), we just need to specify the sample size ($n$) of interest: this could be the available number of participants in an existing dataset being considered for model development or it might be a specified sample size being considered for new data collection (e.g. determined by *pmsampsize* for criteria (i) to (iii)). Usually, a range of different sample sizes will be considered to examine the value of information (e.g. in terms of reduced width of uncertainty intervals, reduced classification instability) arising from including additional participants over and above that recommended by *pmsampsize*.

- Option B: calculate a target sample size for new data collection to ensure precise individual-level predictions

When designing a new study to recruit participants for model development, researchers will want to calculate the sample size required to target particular precision of risk estimates. By rearranging Eq. (6), the sample size needed to target a chosen variance of the logit-risk estimate for an individual is:

9 $$n ={{\text{var}}\left(\text{logit}({p}_{new})\right)}^{-1}{{\varvec{x}}}_{new } {\mathbf{I}}^{-1}{{\varvec{x}}}_{new}^{\prime}$$

We can apply this to every individual in the (real or simulated) dataset from Step (2) to obtain the required $n$ for their particular combination of predictor values (${{\varvec{x}}}_{\text{new}})$. A practical issue is how to select the target value of ${\text{var}}\left(\text{logit}({p}_{\text{new}})\right)$ for each individual, as this is on a difficult scale to interpret. Further, the required value of ${\text{var}}\left(\text{logit}({p}_{\text{new}})\right)$ will not be consistent across individuals, due to the multiplication with ${{\varvec{x}}}_{\text{new} } {\mathbf{I}}^{-1}{{\varvec{x}}}_{\text{new}}^{\prime}$, which is individual-specific. A pragmatic approach is to specify the maximum ${\text{var}}\left(\text{logit}({p}_{\text{new}})\right)$ allowed for a range of ${p}_{\text{new}}$ values (e.g. 0.01, 0.025, 0.05, 0.10, 0.15, 0.20, etc.), corresponding to a target maximum uncertainty interval width on the risk scale (via Eq. (8)). Equation (9) can then be applied to each individual by using the ${\text{var}}\left(\text{logit}({p}_{\text{new}})\right)$ value that corresponds to the categorised ${p}_{\text{new}}$ value closest to their estimated ${p}_{\text{new}}$. We apply this process to the foot ulcer example, later. Special attention may also be given to selecting appropriate ${\text{var}}\left(\text{logit}({p}_{\text{new}})\right)$ values in particular subgroups defined by combinations of predictor values (e.g. sex, ethnicity), where algorithmic fairness checks will be important.

### Deciding and presenting target uncertainty intervals with patients and clinical stakeholders: a perspective based on risk thresholds and decision analysis theory

Regardless of whether options A or B is chosen, model developers will need to decide what width of uncertainty intervals they deem appropriate. This is always context-dependent for the clinical setting of interest; for example, it can depend on patient preferences about potential outcomes, treatments and consequences, and corresponding risk thresholds to inform decision making. This could be discussed with stakeholders (e.g. patients, doctors, health professionals and regulators) in advance of data analysis, to help identify what levels of uncertainty would lead to them recommending further research is still needed [34].

Ideally, a suitably narrow interval is desired for *every *individual (for all combinations of predictor values). However, depending on the clinical context and role of the model for clinical practice, some regions of estimated risk may not require intervals to be as narrow as in other regions. For instance, having wide uncertainty intervals for individuals with high risk (e.g. reflected by uncertainty intervals from 0.3 to 0.95) may not matter if the entire interval range is still compatible with a perceived high risk. This concept aligns with preferences for risk thresholds for clinical decisions. For example, 10-year CVD risk thresholds of 10% are sometimes used to guide decisions to prescribe statins, so wide uncertainty intervals that span 0.3 to 0.95 might be deemed acceptable but narrower intervals that span 0.05 to 0.3 may not. Therefore, when deciding upon appropriate uncertainty interval widths, it is imperative to understand the clinical context of how the model will be used to guide decision making and any corresponding risk threshold(s) involved [35–37]. More discussion of this is given in Supplementary material S2.

In this context, the aim of our sample size approach is to help understand and examine what sample sizes are likely to give sufficient information to guide decisions at the individual level. To help examine this, we recommend calculating and presenting to stakeholders:

- *Prediction instability plots*, where each individual’s ‘true’ risk from Step 3 (*x*-axis) is plotted against their corresponding uncertainty interval (*y*-axis) from Step 5. The question to ask stakeholders is whether the individual uncertainty intervals are too wide for using or endorsing the model in practice. To facilitate this discussion, we recommend prediction instability plots are presented with two curves (e.g. using a LOWESS smoother or spline function) fitted separately through individuals’ upper and lower uncertainty interval values. These curves define a ‘typical’ 95% uncertainty interval at each risk, across the entire spectrum of estimated risks from 0 to 1, which should aid visual interpretation for stakeholders (as individual uncertainty intervals can vary considerably, even for those with the same estimated risk). This is demonstrated later, in our kidney injury application.
- *Classification instability plots (if risk thresholds are relevant)*, plotting each individual’s ‘true’ risk (*x*-axis) (i.e. that risk defined by the ‘core model’) against the proportion (*y*-axis) of their uncertainty distribution that falls on the opposite side of their chosen clinical risk threshold compared to their ‘true’ risk. The question to ask stakeholders is whether, in general, what probability of misclassification (i.e. the proportion of the uncertainty distribution in the opposite direction) is generally too large for them to use or endorse the model for individuals in practice. In our examples later, we assume the same risk threshold is relevant for all individuals, but this can be relaxed if stakeholders recommend different thresholds across particular subgroups.
- *Summary statistics* that quantify the magnitude of uncertainty in key performance and classifications measures of interest across individuals. In particular, the mean (min, max, median, etc.) width of 95% uncertainty intervals for an individual risk and the mean (min, max, median, etc.) probability of individual misclassification. Also, the mean (min, max, median, etc.) across individuals of their mean absolute prediction error (MAPE), which can be derived by many (e.g. 1000) sampling values from each individual’s uncertainty distribution and calculating mean absolute differences to their ‘true’ risk. Instability (uncertainty) in other (more population-level) measures such as calibration slope, net benefit, sensitivity and specificity might also be relevant, though not considered in this article.
- *Subgroup plots and results* that summarise the anticipated uncertainty and classification instability in relevant subgroups of people (e.g. defined by relevant attributes including sex and ethnicity, and other variables important for fairness checks).

## Results I: applied examples

We now consider two examples. The first considers a situation in advance of data collection with the ‘core model’ taken from an existing model being updated. The second considers when an existing dataset is available but less information exists to inform the ‘core model’.

### Example 1: prediction model for foot ulcer

Chappell et al. developed a prediction model for the risk of foot ulcer by 2 years in people with diabetes, [28] which contained three predictors. Let us assume that a new cohort study is planned to collect new data to update and potentially extend this model. What sample size should be targeted? Researchers should start by considering criteria (i) to (iii) (to target population-level stability), for which *pmsampsize* suggests a minimum sample size of 453 participants (27 events), based on an overall risk of 0.059 and three predictor parameters. However, we should also examine the expected uncertainty of individual-level predictions and whether a larger sample size is needed for more precise and stable risk estimates. To examine this, we applied our new five-step process using the decomposition of Fisher's information matrix, as follows.

#### Step 1: Identify core predictors

The core predictors were chosen to be all those included in Chappell’s model: mono (1 if insensitive to monofilament), pulse (1 if missing at least one-foot pulse) and history (1 if previous ulcer or amputation). Although other predictors might be considered in the new model, it was deemed fundamental to examine individual-level uncertainty of risk estimates across combinations of these three predictors.

#### Step 2: specify the joint distribution of the predictors

Upon request, Chappell et al. summarised the joint distribution of these three binary predictors in their model development dataset:

- Mono 0 pulse 0 history 0 (56.3%)
- Mono 1 pulse 1 history 1 (2.1%)
- Mono 1 pulse 1 history 0 (6.2%)
- Mono 1 pulse 0 history 1 (3.2%)
- Mono 1 pulse 0 history 0 (11.5%)
- Mono 0 pulse 1 history 1 (1.2%)
- Mono 0 pulse 1 history 0 (17.7%)
- Mono 0 pulse 0 history 1 (2.0%)

We simulated a dataset of 10,000 participants with predictor values randomly sampled from this joint distribution.

#### Step 3: specify the ‘core model’

The ‘core model’ was assumed to be the same as Chappell et al.:

$$\text{ln}\left(\frac{{p}_{i}}{1-{p}_{i}}\right)=-3.81 + (1.11 \times \text{mono}) + (0.70 \times \text{pulse}) + (1.95 \times \text{history})$$

For every individual in the simulated dataset, we used this model to generate their true (logit) risk conditional on their simulated predictor values from Step 2.

#### Step 4: derive Fisher’s unit information after decomposing Fisher’s information matrix

The unit information matrix was calculated using Eq. (3) (equivalent to Eq. (4) for this three predictor model), by calculating each of the components of $\mathbf{A}$ for each individual in the simulated dataset from step (2) (conditional on their predictor values and ‘true’ risks), and then taking the means (across all participants) of each component to give expected values and thus form $\mathbf{I}$. This is embedded in our software module (see below).

#### Step 5: identify the minimum sample size needed to achieve narrow uncertainty intervals

Finally, options A and B of Step 5 were both applied to examine the sample size required for precise individual-level risk estimates. This required multiple investigations, with consideration of clinical context, as follows.

- (i) Applying option A

Option A can be used to calculate anticipated uncertainty intervals of individual risk based on a user-specified sample size, which is implemented in our accompanying *pmstabilityss* module. It requires the (simulated) dataset from Step 2, the variable names denoting the core predictors (Step 1), and the overall risk, sample size and assumed betas of the ‘core model’ (Step 3). It then implements Step 4 and option A of Step 5, to derive uncertainty intervals for all individuals in the dataset, summary statistics about the uncertainty intervals and MAPE (e.g. mean, minimum and maximum), and prediction and classification instability plots. The full code and explanation of each term are provided in the *pmstabilityss* help file; briefly, here, the Stata code is.

pmstabilityss mono pulse history, prev(0.059) lp(lp_true) nodraw tol(0.001) threshold(0.06) n(453).

where ‘lp_true’ is a variable defined by the ‘core model’ equation in Step 3.

**Fig. 1:**
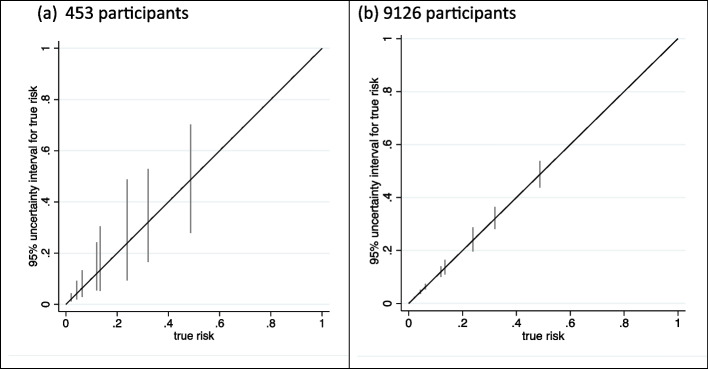
Anticipated uncertainty interval widths when developing a foot ulcer prediction model with a sample size of **a** 453 participants and **b** 9126 participants, using a ‘core model’ based on the prediction model of Chappell et al.

In this example, there are 8 groups of participants (defined by the 8 combinations of values of the 3 binary predictors). Assuming a total sample size of 453 participants (the *pmsampsize* minimum recommended based on criteria (i) to (iii)), anticipated 95% uncertainty intervals for each group are shown by the prediction instability plot of Fig. 1(a). The mean (min, max) interval width is 0.08 (0.03, 0.42) with, as expected, wider intervals for those with less common combinations of predictor values. In particular, individuals with a ‘true’ risk of 0.49 have the least common set of predictor values (mono 0, plus 1, history 1), leading to a wide 95% uncertainty interval of 0.28 to 0.70. The mean (min, max) MAPE is 0.017 (0.0003, 0.20).

Alongside individual-level uncertainty, we can also examine instability in subgroups defined by one particular predictor value (essentially integrated over the distribution of the other two predictors in the model). For example, in those missing at least one foot pulse, the average uncertainty interval width is 0.14 compared to 0.06 for those with no missing foot pulse and the mean MAPE is 0.027 compared to 0.013. Thus, predictions are more uncertain for those missing a foot pulse.

- (ii) Applying option B to target uncertainty interval widths of 0.1 or less

Now, consider using option B to calculate the sample size required to target uncertainty interval widths $\le$ 0.1 for *all* individuals. This can be calculated by applying Eq. (9) and is implemented in *pmstabilityss*; for example, in Stata, the code is.

pmstabilityss mono pulse history, prev(0.059) lp(lp_true) nodraw threshold(0.06) pciwidth(0.1) pcut(1) logitpvarincrement(0.0005)

This calculates that a sample size of 9126 participants is required, which is driven by the group with a risk of 0.49 who, as mentioned, have the least frequent set of predictor values. The corresponding prediction instability plot for 9126 participants is shown in Fig. 1(b) and, as targeted, all interval widths are 0.1 or less.

#### Decision-analysis perspective: identifying the target sample size considering clinical context and classification instability

Although option B sample size of 9126 participants would be ideal to target narrow intervals for everyone, it may not be achievable or even necessary. Consideration of clinical context and decision thresholds is key here. In their paper, Chappell et al. suggest a risk threshold of about 6% for deciding when individuals should be prescribed preventative treatment for foot ulcers. Therefore, an interval width of 0.1 for everyone may be too stringent, especially those individuals with a predicted risk of 0.49, as the 6% threshold is far from their actual risk. Even if their uncertainty interval width was, say, 0.2 or 0.3, then the interval would still be precise relative to the threshold.

**Fig. 2:**
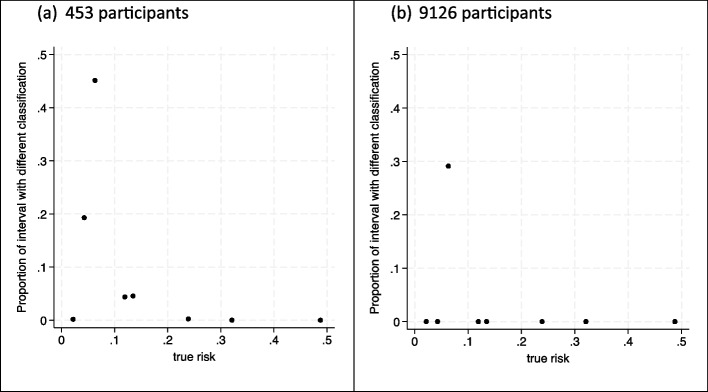
Classification instability plots based on developing the foot ulcer prediction model for (**a**) 453 participants and (**b**) 9126 participants, showing the proportion (y-axis) of each individual’s uncertainty distribution that is on the opposite side of the 6% risk threshold compared to their ‘true’ risk (x-axis)

We can examine this by using a classification instability plot, which *pmstabilityss* produces if the user specifies the threshold value, which here is 6%. Figures 2a and b show classification instability plots for the two sample sizes of 453 and 9126 participants, respectively. They reveal the proportion of each individual’s uncertainty distribution that is on the other side of the threshold compared to their ‘true’ risk (i.e. their probability of misclassification). For individuals with a ‘true’ risk of 0.063 close to the threshold, a high proportion of their uncertainty distribution is on the opposite side of the threshold to their ‘true’ risk. Such uncertainty close to the threshold is inevitable unless extremely large sample sizes are used. However, for other individuals, the classification instability is low even in the minimum sample size of 453 participants recommended by *pmsampsize*. For example, for those with a ‘true’ risk of 0.13, only about 5% of their uncertainty distribution is below the threshold when the sample size is 453 participants and close to 0% with 9126 participants. For either sample size, the instability is close to 0% for individuals with ‘true’ risks close to 0 or above 0.2. Therefore, the sample size of 453 participants (identified from *pmsampsize* based on criteria (i) to (iii)) still seems appropriate to obtain narrow enough uncertainty intervals in the context of a risk threshold of 6%. The value of additional information beyond this appears slight.

**Fig. 3:**
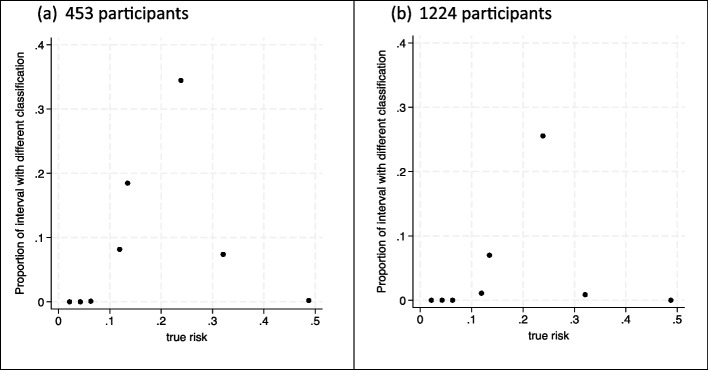
Classification instability plots when developing the foot ulcer prediction model with (**a**) 453 participants and (**b**) 1224 participants, showing the proportion (y-axis) of each individual’s uncertainty distribution that is on the opposite side of the 20% risk threshold compared to their ‘true’ risk (x-axis)

However, what if stakeholders had suggested a threshold of 20% was relevant to consider? For example, it might be that available treatments are very expensive or have side effects that warrant a higher risk threshold for action. This higher threshold may lead to a larger required sample size, because with 453 participants half the individuals still have uncertainty intervals that considerably overlap the 20% threshold (Fig. 1a), and so, the classification instability index is higher than before (Fig. 3a). In particular, we might be concerned about the uncertainty interval width for the individuals with a ‘true’ risk of 0.13 (the mono = 0, pulse = 0, history = 1 group), as their classification instability index is large.

To address this, option B can be used to target a narrower uncertainty interval width of, say, 0.15 (a target ${\text{var}}\left(\text{logit}({\text{p}}_{\text{new}})\right)$ of about 0.103) for this group of people who have a ‘true’ risk of 0.13, and applying Eq. (9) suggests a sample size of 1224 participants. The classification instability index is now much lower (Fig. 3b). Hence, if the threshold of 20% was of interest, researchers may need a sample size of about 1224 participants to target narrow uncertainty intervals and low classification instability for most individuals.

### Summary

This example demonstrates how *pmstabilityss* allows researchers to examine the anticipated precision of individual-level risk estimates, beginning at the sample size recommended by *pmsampsize*, and how the required sample size for assurance can depend heavily on any risk thresholds for decision-making.

### Example 2: prediction of acute kidney injury from an existing dataset

Now, we present an example where an existing dataset (representative of the target population) is already available to researchers who want to examine (and perhaps justify to grant funders) its suitability for developing a model for use in intensive care patients, to estimate an individual’s risk of acute kidney injury within 48 h. The dataset was obtained from the Medical Information Mart for Intensive Care III (MIMIC-III) [38], which contains freely available and de-identified critical care data from the Beth Israel Deaconess Medical Center in Boston, Massachusetts, between 2001 and 2012. A cohort of size 20,413 patients was extracted from this database, containing individuals aged over 18 years of age who were admitted to the intensive care unit for any cause for at least 24 h. Acute kidney injury (the outcome of interest) was defined as present if the maximum creatinine within 48 h after the prediction time (end of first day on ICU) was either (i) more than 1.5 times the minimum day 1 creatinine value or (ii) over 0.3 mg/dL greater than the minimum day 1 creatinine value [39]. The overall risk of acute kidney injury was 17% in the dataset.

The researchers want to ascertain (in advance of any analysis) whether this existing dataset is likely to produce a model with suitably precise individual-level risk estimates and classifications. The five-step process described in our methods section can be used can be used to do this, as follows.

#### Step 1: identify core predictors

There is a general lack of consensus on the key predictors in this field [40], but the existing dataset contained nine variables included in a previous model by Zimmerman et al. [29], so these were selected as the core predictor set (age, sex, bicarbonate, creatinine, haemoglobin, blood urea nitrogen, potassium, systolic blood pressure and SpO_2_).

#### Step 2: specify the joint distribution of the predictors

As the existing dataset was available, the joint distribution of these 9 predictors could be observed directly, and thus, no simulated data was necessary. We simply use the existing dataset at hand.

#### Step 3: specify the ‘core model’

Previous models in this field have been quite poorly reported, with little information on the sizes of predictor effects or the scale that predictors are measured. In this situation, to implement our approach, we focus on a ‘core model’ that corresponds to a previously reported overall risk of 0.174 and *C*-statistic of 0.78 (Zimmerman et al. [29]), whilst making a pragmatic (but potentially strong) assumption about the relative predictor weights:

$$\text{ln}\left(\frac{{p}_{i}}{1-{p}_{i}}\right)=\alpha +\delta \left(1.{\text{age}}_{1i}-1.{\text{male}}_{2i}-1{.\text{bicarbonate}}_{3i}+1.{\text{creatinine}}_{1i}-1{.\text{haemoglobin}}_{2i}-1.{\text{nitrogen}}_{3i}-1.{\text{potassium}}_{1i}-1.{\text{SBP}}_{2i}-1.{\text{SpO}2}_{3i}\right)$$

Here, each of the eight continuous predictors is standardised so that their relative weights are the same (e.g. a 1-SD increase in SBP has the same weight as a 1-SD increase in age) and have the same weight as being male (compared to female). Then, our *pmstabilityss*module implements the iterative process of Austin [33], to identify $\alpha =-1.97$ and $\delta =0.40$ as the required values to ensure the model corresponds to an overall risk of 0.174 and a *C*-statistic of 0.78. Using this model, we calculated true (logit) risks for each of the 20,413 individuals, conditional on their observed predictor values.

#### Step 4: derive Fisher’s unit information after decomposing Fisher’s information matrix

We calculated the unit information matrix ($\mathbf{I}$) using Eq. (3), by calculating each component of $\mathbf{A}$ for each participant in the existing dataset (using their observed predictor values combined with the ‘true’ risks from step (3)) and then taking the means (across all participants) of each component to form the entries of $\mathbf{I}$.

#### Step 5: derive anticipated uncertainty intervals and classification instability

Finally, option A was used to calculate anticipated uncertainty intervals for each of the 20,413 individuals in the existing dataset. Our *pmstabilityss* module calculates this after supplying the existing dataset from Step 2, and the coded variable names representing the core predictors from Step 1 and the ‘core model’ from Step 3 (for full details, see https://github.com/JoieEnsor), via the code:

pmstabilityss age gender bicarbonate creatinine hemoglobin bun potassium sysbp spo2, prev(0.174) cstat(0.78) lp(lp_true) nodraw pmss tol(0.001) threshold(0.1)

where ‘lp_true’ is a variable defined by the ‘core model’ equation in Step 3 and other options are as outlined in the help file.

**Fig. 4:**
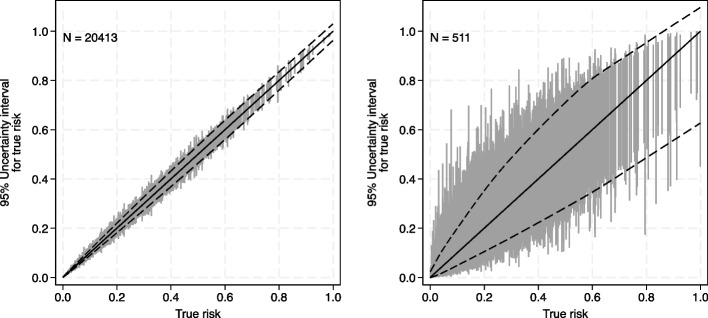
Expected uncertainty interval widths when developing an acute kidney injury prediction model with a sample size of (**a**) 20413 participants (size of existing dataset) and (**b**) 511 participants (minimum size recommended by criteria (i) to (ii))

The prediction instability plot is shown in Fig. 4, displaying all anticipated individual uncertainty intervals and also smooth curves fitted through the lower and upper values of the intervals, to generate ‘typical’ 95% uncertainty intervals for each ‘true’ risk. Regardless of the ‘true’ risk, the uncertainty intervals are all very narrow, which is reassuring to the researchers and potential funders. The mean (min, median, max) uncertainty interval width is 0.027 (0.0006, 0.022, 0.17).

### Decision-analysis perspective

**Fig. 5:**
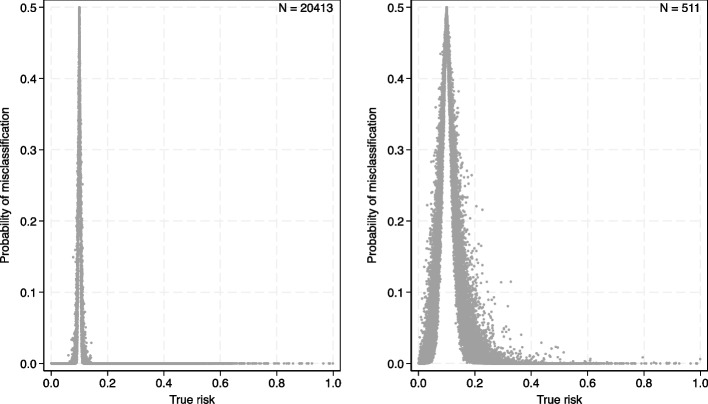
Classification instability plots for the acute kidney injury model when developing a model with (a) (b) 20413 participants (size of existing dataset), or (b) 511 participants (minimum size recommended by criteria (i) to (ii)); the plot shows the proportion (y-axis) of each individual’s uncertainty distribution that is on the opposite side of the 10% risk threshold compared to their ‘true’ risk

Consider that stakeholders suggest a risk threshold of about 10% is relevant for informing clinical decisions, such as closer monitoring of urine output and renal function. The corresponding classification instability plot is shown in Fig. 5, and the instability is close to zero for most individuals, except those very close to the assumed threshold of 10%. The mean (min, median, max) probability of misclassification is 0.022 (0, 0, 0.50). Thus, the existing sample size of 20,413 is likely to be more than sufficient.

### Comparison to smaller sample sizes

For comparison, let us consider the prediction and classification instability plots had the existing dataset matched the sample size of criteria (i) to (iii), which *pmsampsize* calculates to be 511 participants. This is substantially smaller than the 20,413 actually available and leads to wide anticipated uncertainty intervals (Fig. 4b) with larger classification instability (Fig. 5b). The mean (min, median, max) width of uncertainty intervals is 0.17 (0.007, 0.15, 0.81) and the probability of misclassification is 0.13 (0, 0.06, 0.50). ‘Typical’ uncertainty intervals are depicted between the dashed lines; for example, someone who has a ‘true’ risk of 0.2 has a ‘typical’ uncertainty interval from about 0.1 to 0.4. Such wide intervals might reduce model acceptability and usefulness and highlight how the minimum sample size based on criteria (i) to (iii) does not necessarily guarantee precise individual-level risk estimates. Nevertheless, discussions with stakeholders would be needed to properly ascertain whether the magnitude of uncertainty and classification instability would be unacceptable, had the existing sample size been 511 participants.

Supplementary material S3 shows the prediction and classification instability plots had the existing dataset only had 221 participants, the minimum required to estimate the overall risk precisely (criterion i). The uncertainty intervals are now extremely wide, and the classification instability index is quite large for most individuals.

### Summary

This example demonstrates how *pmstabilityss* helps researchers examine the suitability of an existing dataset, in advance of analysis, for producing a model with precise individual-level risk estimates, and how larger sample sizes may be needed than the minimum required by *pmsampsize* for this purpose.

### Results II: an examination of stability in subgroups to inform model fairness checks

The sample size calculation might also examine the anticipated robustness of model predictions in subgroups, as part of fairness checks at the study design stage. To illustrate this, we return to the acute kidney injury example and examine subgroups defined by ethnicity, recorded as a categorical variable, with the following distribution: White (72%), Black (8%), Asian (2%), Hispanic (4%), other (3%) and unknown (11%). In the full dataset of 20,413 participants, this corresponds to 14,774 White, 1584 Black, 508 Asian, 731 Hispanic, 579 other and 2237 unknown. Hence, although the overall sample size and number of Whites are very large, some other ethnic groups are relatively low. This is even more evident in a random sample of 511 participants (the minimum recommended by *pmsampsize*), with about 370 White, 40 Black, 13 Asian, 18 Hispanic, 14 other and 56 unknown. This may raise concerns that any model would be unreliable and imprecise in, for example, Black, Asian and Hispanic groups.

### Ethnicity not included in the ‘core model’

Recall, ethnicity was not an included predictor in the ‘core model’; this will sometimes be deemed appropriate in ‘fear of making clinical decisions race-sensitive’ [41]. Thus, the small sample sizes for some ethnic groups are not relevant in terms of estimating the prognostic effect of ethnicity itself. Rather, the imprecision of risk estimates for each ethnic group will depend on their relationship with the joint distribution of predictors actually included in the ‘core model’. Regardless of the specified total sample size, the precision of estimated risks and classification stability appear similar across ethnic groups. For example, with a total sample size of 511 participants, the mean (min, max) uncertainty interval widths are 0.17 (0.0073, 0.81) for Whites and 0.17 (0.024, 0.68) for Asians.

### Ethnicity included in the ‘core model’

**Fig. 6:**
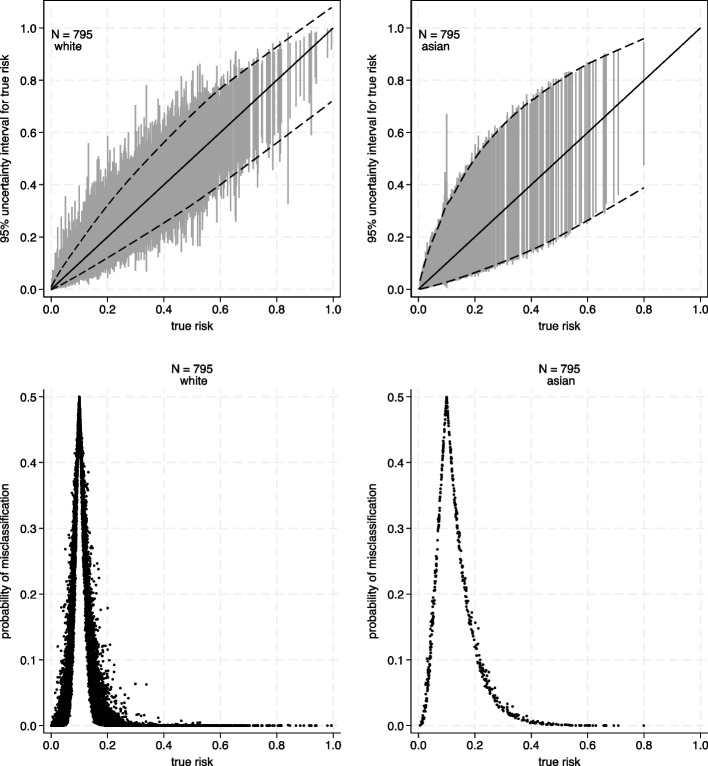
Prediction instability (first row) and classification instability (bottom row) plots for whites (first column) and Asians (second column) in the full dataset for the acute kidney injury model when using 795 participants to develop the model, and assuming a 10% risk threshold, with ethnicity included in the model

Now, let us consider if ethnicity *had* been included as one of the core predictors in the ‘core model’ and that non-White ethnic groups have a higher risk than Whites. Whites are the reference group, and the other five ethnic groups have the same relative weight as other core predictors included in the model. With the additional five parameters, *pmsampsize* now suggests a minimum sample size of 795 participants. At this sample size, the expected uncertainty interval widths and misclassification probabilities are now quite discrepant across ethnic groups (Table 1, Fig. 6); for example, about twice as high for Asians as for Whites. This issue is masked when looking at all participants combined because the majority of the population is White (Table 1). As the sample size is small (795 participants) and a large proportion of the population are White, the non-White ethnic groups have quite sparse data and their estimated risks are far more imprecise than for Whites (Fig. 6).

This demonstrates a tension about developing a model including a predictor with categories that are sparsely represented in the development dataset: though the predictor may help improve discrimination performance, it may lead to larger uncertainty of risk estimates for participants in the sparse category and potentially larger required sample sizes to address this. Indeed, in the original dataset of 20,413 participants, unfairness concerns are diminished due to the much larger sample size. Supplementary material S4 additionally examines males and females.

## Discussion

In this article, we proposed how researchers can examine the potential sample size required to develop or update a prediction model that estimates sufficiently precise and fair individual risks for the clinical context at hand. This is relevant at the study design stage, perhaps as part of a grant application, to help researchers understand whether the minimum recommended by *pmsampsize* (based on population-level stability) is likely also sufficient for individual-level stability and fairness across key subgroups and, if not, by how much the sample size may need to be increased to inform potential costs, timescales and feasibility.

Two examples showcased the approach and our software’s *pmstabilityss*. The approach is most readily applicable when the pilot or existing data are available to inform case-mix distributions and when aiming to update or build from existing models that provide information about (relative) predictor weights, overall risk and *C*-statistic. The approach is not a panacea: like any sample size calculation, it requires assumptions about the underlying truth, which may be difficult to gauge and so require sensitivity analyses across various plausible values. Nevertheless, we hope it encourages researchers to be pro-active, in advance of data collection and model building, about the potential for individual-level stability issues, rather than simply hoping it will be adequate once model development begins. Indeed, grant funders may require some reassurance about achievable sample sizes and the potential corresponding individual-level instability. Once model development begins, internal validation (including prediction and classification instability checks via bootstrapping [20]) and learning curves remain important [30], regardless of the sample size attained.

We also emphasised the importance of considering the actual clinical setting of interest, with input from key stakeholders including health professionals and patients about acceptable uncertainty intervals and, if relevant, classification instability. Currently, such discussions between the model developers and stakeholders are either not done at all or tend to be conducted after model development. We hope our approach will force the communication to begin at the onset of the development study, in the context of the clinical decision to be made. Where risk thresholds are relevant, this can be framed within a decision-theoretic perspective. Sadatsafavi et al. examine this at the population level, with regard to the value of information and net benefit, [42, 43] whereas we considered classification instability (probability of misclassification) at the individual level. Our work is also aiming to improve the fairness of developed prediction models, as it allows researchers to plan and target precise predictions for key subgroups. We recognise precision and instability checks do not cover all aspects of fairness and that precise risk estimates do not necessarily lead to health equity.

Our proposal utilises maximum likelihood estimation theory for standard (unpenalised) logistic regression models and formally examines epistemic uncertainty (*reducible *model-based uncertainty) that arises from fitting a logistic regression with a core set of predictors [44]. We do not consider aleatoric uncertainty (*irreducible* uncertainty) that refers to residual uncertainty that cannot be explained by the ‘core model’. Our decomposition into Fisher’s unit information matrix and the total sample size makes the computations relatively fast as, once the unit information matrix is derived, variances of individual-level risk estimates (and subsequent uncertainty intervals) can be quickly calculated for a range of sample sizes. Using the framework of Heinze et al., [45] we view our approach as phase 3 methodology work for those building (penalised) logistic regression models in situations where overfitting is anticipated to be low. For non-regression machine learning approaches and settings with small data and large numbers of predictors, further research is needed [46, 47], though previous research suggests that instability (and thus required sample size) is often larger for that situation [48].

We suggest a (logistic) regression format for the ‘core model’ as it allows a transparent equation and relative predictor weights to be specified. This is more problematic for black-box approaches, such as random forests; however, to reflect the added complexity from such methods, the regression could include non-linear terms and interactions. We also recognise that (many) other candidate predictors might be considered during model building in addition to the ‘core’ predictors; this will typically add further instability (leading to larger required sample sizes), due to increasing model complexity and overfitting [49], and thus, larger variances of predictions. Further research, including simulation studies, is recommended to examine this and other aspects, including the impact of the assumed ‘core model’ deviating from the truth, and pragmatic approximations such as assuming equal weighting of standardised predictors in the ‘core model’ in the absence of other information.

In summary, we have proposed a decomposition of Fisher’s information matrix to help researchers examine how sample size for model development or updating may impact precision, classification and fairness of individual-level predictions. A companion article (part 2) will extend to time-to-event outcomes and datasets with censoring.

## Supplementary Information

Supplementary Material 1.

## Data Availability

The dataset was obtained from the Medical Information Mart for Intensive Care III (MIMIC-III) trial, which contains freely available and de-identified critical care data from the Beth Israel Deaconess Medical Center in Boston, Massachusetts, between 2001 and 2012. See: Johnson AE, Pollard TJ, Shen L, Lehman LW, Feng M, Ghassemi M, et al. MIMIC-III, a freely accessible critical care database. Sci Data. 2016;3:160035.
