# Supplementary material for: A decomposition of Fisher’s information to inform sample size for developing or updating fair and precise clinical prediction models for individual risk—part 1: binary outcomes
Source: Diagn Progn Res. 2025 Jul 8;9:14. doi: 10.1186/s41512-025-00193-9 (PMC12235806; doi:10.1186/s41512-025-00193-9)
Supplement: Supplementary file 1 — Supplementary Material 1. [file 41512_2025_193_MOESM1_ESM.docx]

**SUPPLEMENTARY MATERIAL**

**S1: Further details of existing sample size calculation**

## Criterion (i): sample size to target a precise estimate of the overall outcome risk

Precision in the overall risk is the first stability level defined by Riley and Collins.^1^ If the sample size is not even large enough to estimate the overall risk precisely at the population level, then it is futile to consider estimating individual-level risks. For a binary outcome, based on the anticipated overall risk ($\hat{\phi}$) and a target absolute margin of error ($\delta$), corresponding to a target 95% confidence interval width of $2\delta$, the minimum required sample size $(n)$ to estimate the overall risk precisely is approximately:^2^

| $n=\left\lceil\left. \left( \frac{1.96}{\delta} \right)^{2}\hat{\phi}(1-\hat{\phi}) \right\rceil\right.$ | Eq. (10) |
| --- | --- |

We generally recommend aiming for $\delta\leq0.05$, and thus confidence interval width $\leq0.1$ but a smaller margin of error may be sensible for low (or high) event risks.

## Criterion (ii): sample size to target small overfitting of predictor effects

Shrinkage (also known as penalisation or regularisation) methods aim to address the problem of overfitting by reducing the variability in a developed model’s predictions such that otherwise extreme predictions (i.e., predicted risks closest to 0 or 1) are pulled towards the overall average.^3-8^ However, there is no guarantee that shrinkage or penalisation methods will fully overcome the problem of overfitting;^4, 9^ and the larger the shrinkage required, the greater concern that model predictions will be miscalibrated.

To address this, our second criterion targets a sample size ($n$) and number of candidate predictor parameters ($P)$ that minimise the problem of overfitted predictor effects.^2, 10, 11^ The calculation is based on theory for regression models, and requires the researcher to pre-specify the number of candidate predictor parameters ($P$), a uniform shrinkage factor ($S$, which we recommend to be $\leq$0.9 so that overfitting is $\leq10\%$), and the anticipated model performance as defined by the Cox-Snell R-squared statistic $(R_{CS}^{2})$.^2, 5, 12^ The minimum sample size ($n$) is then:

| $n=\left\lceil\left. \frac{P}{(S-1) ln\left( 1-\frac{R_{CS}^{2}}{S} \right)} \right\rceil\right.$ | Eq. (11) |
| --- | --- |

The number of parameters ($P$) should, at least, correspond to a core set of predictors known to be important in the field (see Section 3 for more discussion on this). Anticipated values of $R_{CS}^{2}$ can be taken from previous studies (existing models in the same field) or, in the absence of any other information, assuming the value of $R_{CS}^{2}$ corresponds to an $R_{Nagelkerke}^{2}$of 0.15 (i.e. $R_{CS}^{2}=0.15\times max\left( R_{CS}^{2} \right), where max\left( R_{CS}^{2} \right)=1-\left( \phi^{\phi}\left( 1-\phi\right)^{1-\phi} \right)^{2}))$, such that 15% of the total variance is explained.^2^ The value of $R_{CS}^{2}$ can also be derived from a specified *C* statistic.^13^

## Criterion (iii): sample size to target a small optimism in apparent model fit

The final criterion targets a small difference in the developed model’s apparent and optimism-adjusted values of $R_{Nagelkerke}^{2}$ (= $R_{CS}^{2}/\max\left( R_{CS}^{2} \right)$), as this is a fundamental overall measure of model fit.^14, 15^ The apparent $R_{Nagelkerke}^{2}$ value is the model’s observed performance in the same data used to develop the model, whilst the optimism-adjusted $R_{Nagelkerke}^{2}$ value is a more realistic (approximately unbiased) estimate of the model’s fit in the target population. The approach calculates the shrinkage factor that corresponds to an expected optimism of $\delta$ in $R_{Nagelkerke}^{2}$ :^2^

| $S= \frac{R_{CS}^{2}}{R_{CS}^{2}+\delta\max\left( R_{CS}^{2} \right)}$ | Eq. (12) |
| --- | --- |

We suggest $\delta$ is a small value, such as $\leq$ 0.05. The obtained value of $S$ can then be placed into the previous equation from step 2, to calculate the minimum required sample size ($n)$

***S2: Explanation of how risk thresholds are chosen within a decision-theory perspective***

It is helpful to formalise the choice of risk thresholds within a decision analysis framework. Assume that decision is whether to biopsy (two possible actions: yes or no) and there are two possible states (prostate cancer present: yes or no), which creates four possible scenarios (pathways) shown in Figure S1. Assigned to each scenario is a “utility” (U1 to U4), which is a numerical measure that defines a value placed on a given pathway. These are specific to an individual, and essentially measure their preference for each scenario if it were known that the assumed state in that scenario was correct. The values of U1 to U4 are best considered relative to one another. For example, consider an individual expresses their utility of each pathway as: $U1_{i}=100, U2_{i}=5, U3_{i}=0$and $U4_{i}=10$; this means they are expressing the action of biopsy if they do have prostate cancer ($U1_{i}=100$), to be 10 times more important than the action of no biopsy if they do not have prostate cancer ($U4_{i}=10$).

Figure S1 Summarising the four possible pathways that stem from the decision of whether or not to request a biopsy in an individual that may or may not have prostate cancer


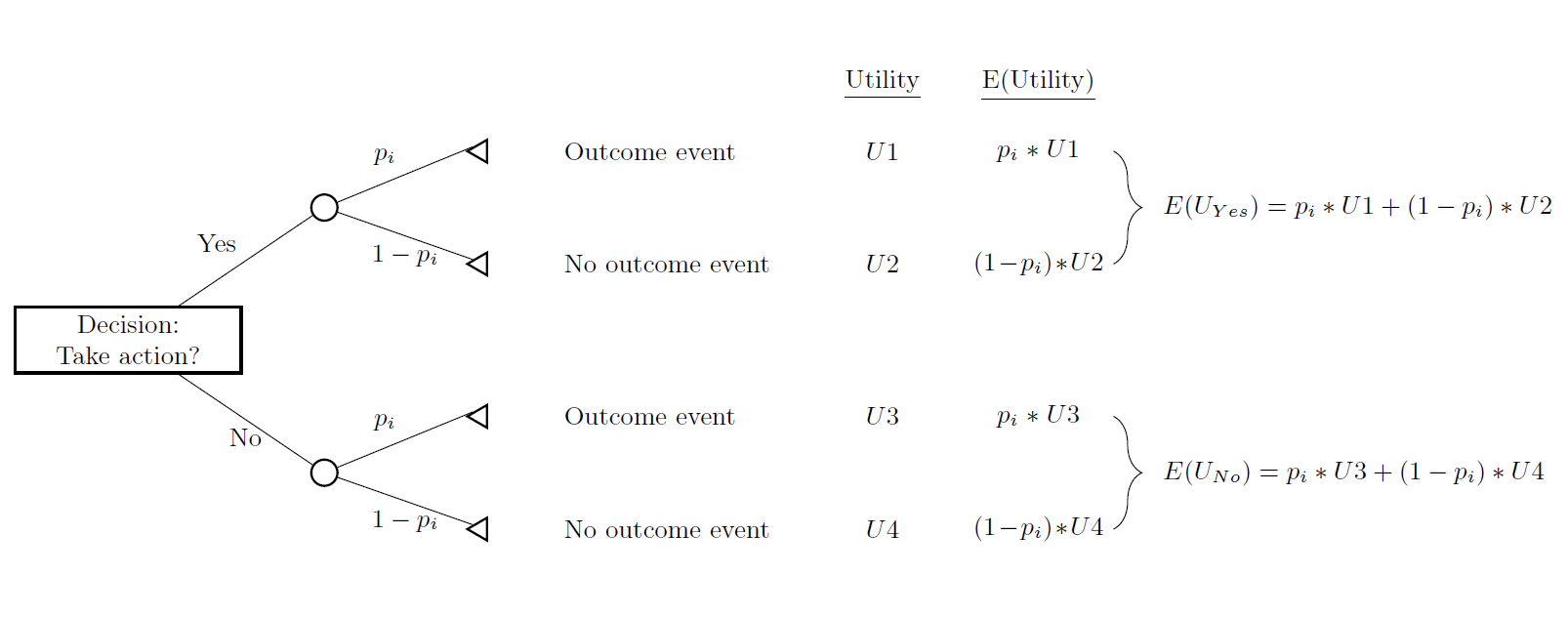


The individual’s chosen utilities also define their risk threshold at which they would be willing to choose a biopsy. It corresponds to where the expected utility from biopsy ($E(U_{Biopsy})$) exceeds the expected utility from no biopsy (e.g., $E(U_{NoBiopsy})$):

$$E(U_{Biopsy})>E(U_{No Biopsy})$$

This can be expressed in terms of the individual’s probability of prostate cancer ($p_{i}$) weighted by the utility values:

$$\left( p_{i}*U1_{i} \right)+\left( \left( 1-p_{i} \right)*U2_{i} \right)>\left( p_{i}*U3_{i} \right)+\left( \left( 1-p_{i} \right)*U4_{i} \right)$$

Rearranging identifies the risk threshold at which the individual prefers a biopsy.^16^

$$p_{\mathrm{THRESHOLD}i} >\left( 1+\frac{U1_{i}-U3_{i}}{U4_{i}-U2_{i}} \right)^{-1}$$

For example, let us return to the individual who expresses their utility of each outcome state as $U1_{i}=100, U2_{i}=5, U3_{i}=0$and $U4_{i}=10$. Then:

$$p_{\mathrm{THRESHOLD}i} >\left( 1+\frac{U1_{i}-U3_{i}}{U4_{i}-U2_{i}} \right)^{-1}=\left( 1+\frac{100-0}{10-5} \right)^{-1}=0.048$$

If a model estimates this individual’s risk of prostate cancer to be $\hat{p}_{i}=0.051$, then this suggests their preference is a biopsy, as their point estimate of risk exceeds their personal risk threshold, and so their expected utility of a biopsy is larger than their expected utility of no biopsy:

$$E(U_{Biopsy})=\left( \hat{p}_{i}*U1_{i} \right)+\left( \left( 1-\hat{p}_{i} \right)*U2_{i} \right)=\left( 0.051*100 \right)+\left( \left( 1-0.051 \right)*5 \right)=9.85$$

$$E\left( U_{No Biopsy} \right)=\left( \hat{p}_{i}*U3_{i} \right)+\left( \left( 1-\hat{p}_{i} \right)*U4_{i} \right)=(0.051*0)+((1-0.051)*10)=9.49$$

Even though this suggests the correct decision is to biopsy, there may still be uncertainty about this decision depending on the precision of $\hat{p}_{i}$. Indeed, understanding the prediction uncertainty may give doctors, health professionals and regulators assurance to use or endorse the model in the first place, or identify when further research is needed. Vickers et al. support this argument,^17^ noting that “decision analysis tells us which decision to make for now, but we may also want to know how much confidence we should have in that decision. If we are insufficiently confident that we are right, further research is warranted.” In this context, the aim of our sample size approach is to help understand and examine which sample sizes are likely to give sufficient information to guide decisions.

***S3: Instability for the acute kidney model with 212 participants (the minimum required to estimate the overall risk precisely)***

| (a)   | (b)   |
| --- | --- |

Figure S2: Expected uncertainty interval widths and classification instability when developing an acute kidney injury prediction model with a sample size of 212 participants, the minimum required to estimate the overall risk precisely

***S4: Instability by sex for the acute kidney injury model***

Recall that sex was a predictor in the ‘core model’. Previously we examined prediction and classification instability plots across all individuals, but now let us consider males and females separately, and assume a risk threshold of 10% is of interest for everyone. Regardless of whether we consider the full sample size of 20413 participants (12186 males, 8227 females) or the smaller sample size of 511 participants (305 males, 206 females), the width of uncertainty intervals and magnitude of classification instability appear quite similar for males and females (figures shown below). Hence, there does not seem to be any strong concerns of a discrepancy in the model’s precision or robustness based on sex, in this example.

**20413 patients: (a) Males (b) Females**

| 1. **Patients: (a) Males (b) Females**    |
| --- |

Figure S3: Prediction and classification instability plots by sex for the acute kidney injury model with 20431 and 511 participants

**S5: Comparison of uncertainty intervals for various modelling approaches**

Our proposal is theoretically driven by Fisher’s information matrix based on unpenalised logistic regression models; it is thus based on exactly the standard errors of individual predictions that would be obtained from a fitted logistic regression model with the same unit information matrix as assumed for the ‘core model’. However, the question remains whether the approach is still relevant for other model development methods are used. Further research is needed, but various approaches are compared for the foot ulcer and kidney examples, below.

1. **Uncertainty intervals for the Chappell example for various modelling approaches**

Let us return to the Chappell example and consider empirically-based uncertainty intervals from a model developed using a random forest, rather than logistic regression, for the identified sample size of 1224 participants. To devise the empirical-based uncertainty interval, we developed 1000 models each trained on three predictors and a random sample of 1224 participants (sampled from the full synthetic data generated in steps 1 to 3 in section 4), with outcome values also randomly generated for each individual based on their assumed ‘true’ risk as specified by the core model in Step 3. Each forest was allowed 100 trees with tree depth of 3, and we applied each of the 1000 models to each participant in the full synthetic dataset to obtain 1000 estimated risks for every individual. The 2.5% and 97.5% percentile values were then used to derive a 95% uncertainty interval for each individual’s risk, as shown in Figure(a). These are considerably wider than the intervals based on the logistic regression (Figure (b)). Varying the number of trees did not affect the findings in this example. For comparison, the empirical-based uncertainty intervals are shown for an unpenalised logistic regression model in Figure (b), which are identical to those from our sample size approach (Figure (d)), as should be anticipated given the theory is based on this approach. The intervals are also very similar to those from a logistic regression with a lasso penalty (Figure(c)), again as might be expected given that criteria (ii) and (iii) aim to minimise overfitting such that penalised and unpenalised approaches are similar.

Next, compare empirically-based uncertainty intervals for the acute kidney injury example and various model development approaches (Figure S4). Again, the interval widths from an unpenalised logistic regression are practically identical to those from our sample size calculation, as expected, and those from the lasso are also very similar. The intervals from the random forest are much larger when using the default software settings for number of trees and depth. There also appears to be concerns of miscalibration.

| (a) random forest   | (b) unpenalised logistic regression   |
| --- | --- |
| (c) lasso logistic regression   | 1. based on sample size calculation    |

Figure S4: Empirical-based uncertainty intervals for individual risks from 1000 models of foot ulcer risk developed using 1224 participants and either (a) a random forest (100 trees and depth of 3), (b) an unpenalised logistic regression, and (c) a logistic regression with lasso penalty. Uncertainty intervals are obtained from 2.5% and 97.5% percentiles of each individual’s 1000 predictions from the 1000 models. For comparison, panel (d) shows the uncertainty intervals derived from our sample size calculation based on the same unit information as the fitted models, and thus give identical results to panel (b).

1. **Uncertainty intervals for the kidney example for various modelling approaches**

| (a) random forest   | (b) unpenalised logistic regression   |
| --- | --- |
| (c) lasso logistic regression   | 1. based on sample size calculation    |

Figure S5: Empirical-based uncertainty intervals for individual risks from 1000 models of acute kidney injury risk developed using 511 participants and either (a) a random forest (default software settings), (b) an unpenalised logistic regression, and (c) a logistic regression with lasso penalty Uncertainty intervals are obtained from 2.5% and 97.5% percentiles of each individual’s 1000 predictions from the 1000 models. For comparison, (d) shows the uncertainty intervals derived from our sample size calculation.

Reference List

1. Riley RD, Collins GS. Stability of clinical prediction models developed using statistical or machine learning methods. Biom J. 2023;65(8):e2200302.

2. Riley RD, Snell KI, Ensor J, Burke DL, Harrell FE, Jr., Moons KG, et al. Minimum sample size for developing a multivariable prediction model: Part II - binary and time-to-event outcomes. Stat Med. 2019;38(7):1276-96.

3. Pavlou M, Ambler G, Seaman SR, Guttmann O, Elliott P, King M, et al. How to develop a more accurate risk prediction model when there are few events. BMJ. 2015;351:h3868.

4. Van Houwelingen JC. Shrinkage and penalized likelihood as methods to improve predictive accuracy. Statistica Neerlandica. 2001;55:17-34.

5. Van Houwelingen JC, Le Cessie S. Predictive value of statistical models. Stat Med. 1990;9(11):1303-25.

6. Copas JB. Regression, Prediction and Shrinkage. Journal of the Royal Statistical Society Series B (Methodological). 1983;45(3):311-54.

7. Tibshirani R. Regression shrinkage and selection via the lasso. J Royal Statist Soc B. 1996;58:267-88.

8. Copas JB. Using regression models for prediction: shrinkage and regression to the mean. Stat Methods Med Res. 1997;6(2):167-83.

9. Van Calster B, van Smeden M, De Cock B, Steyerberg EW. Regression shrinkage methods for clinical prediction models do not guarantee improved performance: Simulation study. Stat Methods Med Res. 2020;29(11):3166-78.

10. Riley RD, Snell KIE, Ensor J, Burke DL, Harrell FE, Jr., Moons KGM, et al. Minimum sample size for developing a multivariable prediction model: Part I - Continuous outcomes. Stat Med. 2019;38(7):1262-75.

11. Harrell FE, Jr., Lee KL, Mark DB. Multivariable prognostic models: issues in developing models, evaluating assumptions and adequacy, and measuring and reducing errors. Stat Med. 1996;15(4):361-87.

12. Cox DR, Snell EJ. The Analysis of Binary Data (second edition). London: Chapman and Hall; 1989.

13. Riley RD, Van Calster B, Collins GS. A note on estimating the Cox-Snell R(2) from a reported C statistic (AUROC) to inform sample size calculations for developing a prediction model with a binary outcome. Stat Med. 2021;40(4):859-64.

14. Harrell FE, Jr. Regression Modeling Strategies: With Applications to Linear Models, Logistic and Ordinal Regression, and Survival Analysis (Second Edition). New York: Springer; 2015.

15. Nagelkerke N. A note on a general definition of the coefficient of determination. Biometrika. 1991;78:691-2.

16. Kaplan J. Decision Theory and the Factfinding Process. Stanford Law Review. 1968;20(6):1065-92.

17. Vickers AJ, Van Claster B, Wynants L, Steyerberg EW. Decision curve analysis: confidence intervals and hypothesis testing for net benefit. Diagn Progn Res. 2023;7(1):11.
